# Bridging cultural gaps through consumption: how social media and perceived value shape international students’ purchase intentions in China

**DOI:** 10.3389/fpsyg.2026.1841619

**Published:** 2026-05-29

**Authors:** Qin Zhang, XueNi Hng, DeZhi Han

**Affiliations:** College of Management and Economics, Tianjin University, Tianjin, China

**Keywords:** cross-cultural consumption adaptation, cultural identity, emotional value, purchase intention, social media

## Abstract

**Introduction:**

With the rapid growth of the international student population in China, understanding their cross- cultural consumption behavior has become increasingly important. However, existing studies have primarily focused on immigrants or long- term residents, leaving the psychological transformation process from value perception to consumption integration among short- term international students largely underexplored.

**Methods:**

This study integrates perceived value theory, cultural adaptation theory, social identity theory, and the theory of planned behavior to construct a dual- mediation and dual- moderation framework. Survey data were collected from international students studying in China, yielding 450 valid responses. Structural equation modeling was used to test the proposed hypotheses.

**Results:**

The results show that: (1) product value and service value influence purchase intention through two parallel mechanisms – an adaptation- based pathway (value → cross- cultural consumption adaptation → cultural identity → purchase intention) accounting for over 50% of the total mediating effect, and an emotional pathway (value → emotional value → purchase intention) explaining approximately 35%; (2) cross- cultural consumption adaptation has a strong effect on cultural identity *β* = 0.553, *p* < 0.001), indicating that cultural adaptation is a key prerequisite for identity formation; (3) social media usage significantly moderates both pathways, with high- frequency users demonstrating greater efficiency in translating perceived value into purchase intention.

**Conclusion:**

This study extends sociocultural adaptation theory to the consumption context by conceptualizing cross-cultural consumption adaptation as a multidimensional construct encompassing cognitive, emotional, and behavioral adaptation to host-country consumption practices. The study develops a three-dimensional measurement framework, identifies both adaptation-based and emotional mediating mechanisms, and reveals the dual moderating role of social media. Practically, the findings provide empirical evidence for firms to design differentiated cross-cultural marketing strategies and for universities to develop support systems that facilitate international students’ cultural adaptation. Limitations include the use of cross-sectional data and potential sample representativeness issues. Future research could employ longitudinal or experimental designs to further validate the proposed mechanisms.

## 1 Introduction

The rapid development of globalization and the internationalization of higher education has significantly reshaped global educational mobility. In recent decades, China has emerged as one of the most important destinations for international students. According to statistics from the Ministry of Education of China, the number of international students studying in China reached more than 490,000 before the COVID-19 pandemic, originating from nearly 200 countries and regions. This large and culturally diverse group not only contributes to the internationalization of Chinese higher education but also forms a rapidly growing consumer market in China. International students participate in various forms of daily consumption, including food, transportation, entertainment, and online shopping, making them an important bridge connecting cultural exchange and economic interaction between China and the world (OECD, 2022^1^).

Consumption behavior plays a crucial role in the process of cross-cultural adaptation. For international students, everyday consumption activities represent not only economic decisions but also cultural learning experiences. According to cultural adaptation theory, individuals encountering a new cultural environment must balance the maintenance of their original cultural identity with adaptation to the host culture (Berry, 1997). Consumption settings often become key arenas where such cultural negotiation occurs. International students in China face multiple cultural differences in the marketplace, including language barriers, unfamiliar payment systems, different service standards, and distinct consumption values (Taiwo and Ofosu-Budu, 2024). As a result, their consumption behavior reflects a gradual process of understanding, adjusting to, and integrating into the host country’s consumption culture (Cleveland and Laroche, 2007).

Existing research on cross-cultural consumption has primarily focused on immigrants or long-term residents (Cleveland et al., 2009). However, international students represent a unique group characterized by short-term residence, temporary cultural contact, and goal-oriented adaptation. Their cross-cultural consumption experiences may therefore differ substantially from those of permanent migrants (Hu et al., 2026). In particular, how international students transform their initial value perceptions of products and services into cultural identification and purchase intentions remains insufficiently explored. Understanding this psychological transformation process is important for both theoretical development and practical applications in cross-cultural marketing and international education management (Zhang and Goodson, 2011).

Meanwhile, the rapid development of digital media has profoundly transformed cross-cultural communication and consumption behavior. Social media platforms such as WeChat, TikTok (Douyin), and Xiaohongshu have become central channels through which international students obtain information, build social connections, and make consumption decisions in China (Kaplan and Haenlein, 2010). Compared with traditional media, social media provides interactive, visual, and emotionally engaging content that helps reduce language barriers and facilitates cultural understanding (Chen and Billedo, 2025). Through user-generated content, influencer recommendations, and algorithm-based information dissemination, social media may accelerate international students’ learning of local consumption norms and enhance their emotional experiences in the host country (Mangold and Faulds, 2009).

Despite the growing importance of social media in cross-cultural contexts, the mechanisms through which social media influences international students’ consumption adaptation remain underexplored. Most existing studies examine the impact of social media on consumer behavior from single perspectives such as information sharing or social interaction, while neglecting its potential moderating role in the cultural adaptation process. In particular, little research has examined how social media simultaneously affects both adaptation-based and emotional pathways that link value perception to cultural identity and purchase intention (Hajli, 2015; Hu et al., 2026).

To address these gaps, the present study investigates the psychological mechanisms underlying international students’ consumption behavior in China. Specifically, this research examines how perceived product value and service value influence purchase intention through two parallel mediating mechanisms: cross-cultural consumption adaptation and emotional value. In addition, the study explores the moderating role of social media usage in strengthening these mechanisms and considers the influence of cultural distance across different student groups.

Using survey data collected from 450 international students studying in China, this study employs structural equation modeling to test the proposed conceptual framework. By integrating perceived value theory (Sweeney and Soutar, 2001), cultural adaptation theory (Berry, 1997), social identity theory (Tajfel et al., 1979), and the theory of planned behavior (Ajzen, 1991), the research develops a comprehensive model that explains how international students gradually move from value perception to cultural identification and ultimately to purchase intention.

This study contributes to the literature in several ways. First, it introduces the concept of cross-cultural consumption adaptation into the consumer behavior literature and applies Berry’s cultural adaptation theory to the consumption context. A multidimensional measurement framework consisting of cognitive, emotional, and behavioral adaptation is developed to capture international students’ integration into the host country’s consumption culture. Second, the study proposes an integrated framework incorporating dual mediating and dual moderating mechanisms. Specifically, it identifies two parallel pathways, a adaptation-based pathway through cross-cultural consumption adaptation and a parallel emotional pathway through emotional value while also examining the dual moderating role of social media across these mechanisms. Third, this research focuses on international students as a unique cross-cultural consumer group. Unlike immigrants or long-term residents, international students experience temporary and goal-oriented cultural contact. By examining this group in the Chinese context, the study provides new empirical evidence for understanding cross-cultural consumption behavior in emerging markets and expands the contextual boundary of cross-cultural consumer research (Taiwo and Ofosu-Budu, 2024).

## 2 Literature review

With the acceleration of globalization and the continuous growth of international education mobility, cross-cultural consumer behavior has become an increasingly important research topic in marketing and consumer behavior studies (Hofstede, 2001). International students represent a unique consumer group because they simultaneously experience cultural transition, identity reconstruction, and consumption adaptation in the host country (Cleveland and Laroche, 2007). Their purchasing decisions are not only influenced by economic and functional considerations but also by cultural values, emotional experiences, and social interactions (Steenkamp, 2019).

Previous studies on consumer behavior have extensively examined the role of perceived value, cultural identity, and emotional responses in shaping purchase intention (Zeithaml, 1988; Stets and Burke, 2000). However, in cross-cultural contexts, consumption behavior becomes more complex because consumers must adapt to unfamiliar cultural norms, languages, and market environments (Sweeney and Soutar, 2001). International students often experience a process of cultural learning and adaptation that influences how they perceive products, interpret services, and construct their consumption preferences (Berry, 1997).

In addition, the rapid development of social media has significantly transformed the way individuals acquire information and interact with cultural environments. Social media platforms provide international students with opportunities to learn about local consumption practices, share experiences, and build social connections with the host society (Cao et al., 2024; Hofhuis et al., 2023; Kaplan and Haenlein, 2010). Through these interactions, students may gradually develop emotional attachment and cultural identification with the host country, which may ultimately influence their purchase intentions (Hofstede, 2001).

Although previous research has examined perceived value and cultural identity separately, limited studies have integrated these perspectives to explore the mechanisms through which perceived value influences international students’ purchase intentions. While the role of social media in intercultural adaptation has received meaningful attention (Akter et al., 2024; Rehman et al., 2024), how social media moderates the relationship between perceived value and consumption adaptation remains underexplored. Similarly, although food acculturation among international students has been studied in relation to buying decisions (Tirelli et al., 2013), a framework integrating perceived value, acculturation, and purchase intention is still lacking. Hu et al. (2026) also noted the need to incorporate perceived value into acculturation frameworks when discussing the dual effects of social media on cross-cultural psychology. Therefore, this study integrates perceived value theory, acculturation theory, social identity theory, and the theory of planned behavior to develop a comprehensive framework for understanding international students’ consumption behavior in cross-cultural environments (Cleveland and Laroche, 2007).

### 2.1 Cultural identity and purchase intention

Cultural identity refers to the psychological sense of belonging, emotional attachment, and cognitive recognition that individuals develop toward a specific cultural group (Stets and Burke, 2000; Shavitt et al., 2006). It reflects the extent to which individuals internalize cultural values, traditions, and social norms as part of their self-concept. Cultural identity is not static, rather, it is continuously shaped and reconstructed through social interactions and cultural experiences (Cleveland and Laroche, 2007).

In consumer behavior research, cultural identity has been widely recognized as an important factor influencing consumer attitudes and purchase decisions. When consumers identify strongly with a particular culture, they are more likely to prefer products and services associated with that culture because such consumption allows them to express their cultural values and social identity (Shavitt et al., 2006). Empirical research has provided strong evidence supporting the relationship between cultural identity and consumption behavior. For example, Liu and Zhao (2024) found that the cognitive, emotional, and evaluative dimensions of cultural identity significantly influence consumers’ willingness to purchase cultural products. Among these dimensions, emotional identification often plays the most important role because emotional attachment strengthens consumers’ psychological connection with culturally meaningful products.

Similarly, Li et al. (2021) found that emotional identification and perceived quality jointly influence consumers’ purchase intentions for culturally symbolic products. When consumers perceive that a product reflects their cultural values and identity, they are more likely to develop positive attitudes toward the product and show stronger purchase intentions. Other studies suggest that cultural identity may also influence consumption indirectly through various mediating mechanisms such as brand trust, symbolic value, and perceived authenticity (Haiyang and Jiaxun, 2017; Zhang and Goodson, 2011). Oyedele (2025) found that when consumers perceive a strong alignment between their cultural identity and a brand’s cultural meaning, they are more likely to develop emotional attachment and loyalty toward the brand.

For international students living in a foreign cultural environment, cultural identity may gradually evolve through exposure to the host culture. As students learn about local traditions, participate in cultural activities, and interact with local communities, they may develop a stronger sense of identification with the host culture. This process of cultural identification may encourage them to engage more actively in local consumption practices and increase their willingness to purchase local products and services. Therefore, cultural identity is expected to have a direct positive effect on international students’ purchase intentions for host-country products.

*H1*: Cultural identity has a significant positive effect on purchase intention.

### 2.2 Direct effects of product value and service value

Perceived value theory suggests that consumers evaluate products and services based on the trade-off between perceived benefits and perceived costs (Zeithaml, 1988). This evaluation process plays a central role in consumer decision-making because perceived value influences attitudes, satisfaction, and behavioral intentions. Sweeney and Soutar (2001) has conceptualized perceived value as a multidimensional construct that includes functional value, emotional value, and social value. In cross-cultural consumption contexts, perceived value becomes particularly important because international consumers often face higher levels of uncertainty and unfamiliarity when interacting with foreign markets.

In this study, product value refers to the international student’s evaluation of the utility derived from goods in the host country’s marketplace. Following Sheth et al. (1991) and Sweeney and Soutar (2001), product value consists of two dimensions: functional value (e.g., quality, durability, performance) and symbolic value (e.g., cultural meanings, identity expression, prestige). Service value refers to the evaluation of utility derived from service encounters. Based on Parasuraman et al. (1988) and Brady and Cronin (2001), service value in cross-cultural contexts comprises efficiency (e.g., speed, convenience, reliability) and cultural friendliness (e.g., warmth, helpfulness, cultural sensitivity). Because international students may encounter language barriers and cultural misunderstandings, they often rely heavily on service interactions to navigate the consumption environment.

#### 2.2.1 Effects on cross-cultural consumption adaptation

Acculturation theory explains how individuals adapt to a new cultural environment through learning and behavioral adjustment (Berry, 1997). National cultural frameworks, such as those proposed by Hofstede (2001) and Steenkamp (2001), provide a macro-level context for understanding why international students from different backgrounds may experience consumption adaptation differently. In consumption contexts, this adaptation process can be conceptualized as cross-cultural consumption adaptation, which refers to the process through which individuals gradually understand and adopt the consumption norms and practices of the host culture. Previous studies have shown that immigrants and international students gradually develop familiarity with local markets and consumption practices through repeated interactions and experiences (Cleveland and Laroche, 2007; Peñaloza, 1994).

When international students perceive high product value, they are more likely to overcome cultural barriers and explore local consumption opportunities. Functional value reduces perceived risk by assuring product quality and reliability, while symbolic value provides cultural meaning that helps students interpret and appreciate host-country products. Similarly, high service value particularly cultural friendliness directly reduces the anxiety associated with cross-cultural service encounters. Efficient and culturally sensitive service interactions create positive reinforcement, encouraging students to engage more frequently with local businesses. Over time, these repeated positive experiences facilitate cognitive understanding, emotional comfort, and behavioral adoption of local consumption practices. Therefore, both product value and service value should positively influence cross-cultural consumption adaptation.

*H2a*: Product value has a significant positive effect on cross-cultural consumption adaptation.

*H2b*: Service value has a significant positive effect on cross-cultural consumption adaptation.

#### 2.2.2 Effects on emotional value

Emotional value refers to the feelings or affective states generated by consumption experiences (Sweeney and Soutar, 2001). Unlike the cognitive dimension of product evaluation, emotional value captures the extent to which a product or service makes the consumer feel good, happy, or relaxed. In cross-cultural contexts, emotional value is particularly salient because international students often experience stress, uncertainty, and culture shock. Positive consumption experiences can mitigate these negative emotions and foster a sense of psychological comfort.

Product value contributes to emotional value in two ways. Functional value provides reliability and reduces anxiety about product failure, leading to peace of mind. Symbolic value allows students to express their emerging affinity for the host culture, generating pride and positive self-conscious emotions. Service value contributes to emotional value primarily through cultural friendliness. When service providers are warm, respectful, and accommodating, international students feel welcomed and valued, which directly enhances positive affect. Efficient service also reduces frustration and saves time, leading to greater satisfaction and enjoyment. Consequently, both product value and service value are expected to increase emotional value.

*H2c*: Product value has a significant positive effect on emotional value.

*H2d*: Service value has a significant positive effect on emotional value.

### 2.3 Emotional value and purchase intention

In addition to functional benefits, consumption experiences often generate emotional responses that influence consumer behavior. Emotional value refers to the affective benefits that consumers obtain from consumption experiences, such as pleasure, enjoyment, excitement, or relaxation (Sweeney and Soutar, 2001). Babin et al. (1994) suggested that emotional value plays a crucial role in shaping consumer attitudes because emotional experiences create psychological satisfaction that goes beyond functional utility. When consumers experience positive emotions during consumption, they are more likely to develop favorable attitudes toward the product and exhibit stronger purchase intentions.

For international students living in a foreign cultural environment, emotional value may be particularly important. Consumption activities may serve as opportunities to explore new cultural experiences, reduce feelings of isolation, and create enjoyable memories in the host country. Positive emotional experiences associated with consumption may therefore strengthen students’ willingness to engage in further purchasing behavior. Furthermore, emotional value can help reduce uncertainty and anxiety associated with cross-cultural environments. When international students perceive that a product or service provides enjoyable emotional experiences, they may feel more comfortable interacting with the local market and more willing to repeat the consumption behavior.

Previous research has also found that emotional value significantly predicts purchase intention in various consumption contexts, including tourism, retail, and cultural products (Babin et al., 1994; Sweeney and Soutar, 2001). Emotional satisfaction enhances consumers’ attachment to products and increases their likelihood of making purchase decisions. Therefore, emotional value is expected to positively influence international students’ purchase intentions.

*H3*: Emotional value has a significant positive effect on purchase intention.

### 2.4 Cross-cultural consumption adaptation and cultural identity

Cross-cultural consumption adaptation refers to the process through which international students gradually adjust their consumption cognition, emotions, and behaviors to the host country’s consumption environment. As conceptualized in this study, it encompasses three dimensions: cognitive adaptation (understanding local consumption norms), emotional adaptation (feeling comfortable during consumption), and behavioral adaptation (adopting local practices).

Cultural identity, as defined earlier, is the sense of belonging and emotional attachment to a cultural group. The relationship between cross-cultural consumption adaptation and cultural identity can be understood through the logic of experiential learning. When international students successfully adapt their consumption behaviors, for example, by learning where to shop, what products are culturally appropriate, and how to interact with local service providers they accumulate firsthand experiences with the host culture. These repeated, positive marketplace interactions serve as tangible evidence of their growing competence and belonging. Over time, cognitive understanding of consumption norms translates into internalized cultural knowledge, emotional comfort fosters positive affect toward the host culture, and behavioral participation reinforces a sense of integration. Consequently, higher levels of cross-cultural consumption adaptation are expected to be positively associated with stronger identification with the host culture.

Empirical support for this logic can be found in acculturation research. Ward and Kennedy (1999) demonstrated that sociocultural adaptation (behavioral competence) is positively associated with psychological adaptation (emotional well-being and identity). In consumer contexts, Askegaard et al. (2005) showed that as immigrants become more adept at navigating local marketplaces, they gradually develop hybrid or host-culture identities. Thus, we propose:

*H4*: Cross-cultural consumption adaptation has a significant positive effect on cultural identity.

### 2.5 The mediating role of cross-cultural consumption adaptation (product/service value → adaptation → cultural identity)

Having established the direct effects of product value and service value on cross-cultural consumption adaptation (H2a, H2b) and the direct effect of adaptation on cultural identity (H4), we now propose a mediation chain. According to acculturation theory, perceived value in the host country’s marketplace serves as an initial stimulus that motivates individuals to engage with the local consumption environment. When international students perceive that local products offer functional and symbolic benefits (product value) and that local services are efficient and culturally friendly (service value), they are more likely to initiate and sustain consumption-related learning and behavioral adjustments. This process of cross-cultural consumption adaptation, in turn, enhances their sense of belonging and emotional attachment to the host culture, that is, their cultural identity. In other words, the effect of perceived value on cultural identity is transmitted through the adaptive experiences that students undergo in the marketplace.

Prior research supports such indirect pathways. For example, Cleveland and Laroche (2007) found that acculturation mediates the relationship between cultural exposure and consumption outcomes. Similarly, Srite and Karahanna (2006) demonstrated that cultural adaptation processes carry the influence of value perceptions onto identity-related outcomes. Therefore, we hypothesize:

*H5a*: Cross-cultural consumption adaptation positively mediates the relationship between product value and cultural identity.

*H5b*: Cross-cultural consumption adaptation positively mediates the relationship between service value and cultural identity.

### 2.6 The mediating role of emotional value (product/service value → emotional value → purchase intention)

As argued in Sections 2.2.2 and 2.3, product value and service value directly enhance emotional value, and emotional value directly increases purchase intention. Combining these two relationships suggests a mediation model: product value and service value may influence purchase intention not only directly but also indirectly through the positive emotions they generate during consumption.

The logic is straightforward. When international students perceive that a product offers functional reliability or symbolic meaning (product value), they experience positive affect such as satisfaction, pride, or enjoyment. Similarly, when they encounter efficient and culturally friendly services (service value), they feel welcomed, relaxed, and valued. These positive emotional states collectively termed emotional value, create an affective bond with the host country’s marketplace. Consumers who feel good about their consumption experiences are more likely to form strong purchase intentions, as positive emotions reduce perceived risk and increase approach behaviors (Babin et al., 1994; Zeithaml, 1988). Thus, emotional value serves as an affective mechanism linking perceived value to purchase intention.

Empirical evidence from cross-cultural consumer research supports this mediation. Sweeney and Soutar (2001) found that emotional value mediates the relationship between product quality and behavioral intentions. In the context of international students, similar indirect effects are plausible. Accordingly, we propose:

*H5c*: Emotional value mediates the relationship between product value and purchase intention.

*H5d*: Emotional value mediates the relationship between service value and purchase intention.

### 2.7 The moderating role of social media use

The development of social media has transformed the way individuals acquire information, interact with others, and participate in cultural exchange. Social media platforms enable users to create and share content, communicate with others, and access information about products and services in real time (Kaplan and Haenlein, 2010). For international students, social media serves as an important tool for learning about the host country’s culture and consumption practices. Platforms such as WeChat, TikTok, and Xiaohongshu provide information about local products, restaurants, entertainment venues, and cultural activities (Cao et al., 2024).

Research has shown that social media can significantly influence consumer attitudes and purchase decisions because it provides social proof, peer recommendations, and experiential information (Hajli, 2015). Through social media interactions, individuals may develop trust in products and brands and gain confidence in their consumption decisions (Hu and Zhu, 2022). In cross-cultural contexts, social media also facilitates cultural learning and identity construction. By observing and participating in online communities, international students can better understand local cultural norms and consumption behaviors. This process may strengthen the relationship between perceived value and consumption outcomes.

**Figure 1:**
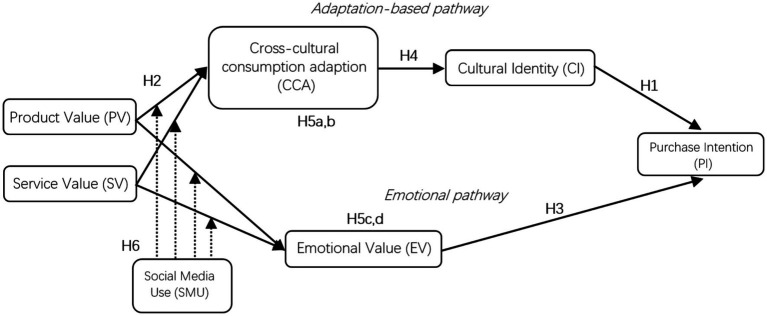
Conceptual model.

Specifically, we argue that social media use is positively associated with stronger relationships between product value and service value and both cross-cultural consumption adaptation and emotional value. When international students actively use social media, they gain access to user-generated content (reviews, unboxing videos, cultural tips) that reinforces their initial value perceptions. For example, a student who perceives high product value in a local brand may, after seeing positive reviews on Xiaohongshu, feel more confident to adapt their consumption behavior. Similarly, social media exposure to culturally friendly service encounters can intensify the emotional value derived from actual service experiences. Therefore, social media use is expected to enhance which positively moderate these relationships (Figure 1).

*H6a*: Social media use positively moderates the relationship between product value and cross-cultural consumption adaptation.

*H6b*: Social media use positively moderates the relationship between service value and cross-cultural consumption adaptation.

*H6c*: Social media use positively moderates the relationship between product value and emotional value.

*H6d*: Social media use positively moderates the relationship between service value and emotional value.

## 3 Methodology

### 3.1 Sampling and data collection

This study adopts a quantitative research design to examine the influence of social media use on the cross-cultural consumption behavior of international students studying in China. A structured questionnaire survey was employed to collect empirical data, which enables statistical testing of the proposed theoretical model and causal relationships among the variables.

The target population consisted of international degree-seeking students enrolled in universities in mainland China. Considering the geographical distribution of international students, the study focused on three major cities with large international student populations: Beijing, Shanghai, and Guangzhou. A stratified convenience sampling approach was applied to improve sample representativeness across different regions and educational levels.

Data collection was conducted from October to December 2024 using both online and offline survey methods. Online questionnaires were distributed through the platform Wenjuanxing and shared through international student social media groups and university international offices. Offline surveys were administered by trained research assistants on university campuses and in international student dormitories.

A total of 564 questionnaires were distributed. After removing responses with abnormal completion times, missing values, repetitive submissions, or inconsistent answers to reverse-coded items, 450 valid questionnaires were retained for analysis, yielding an effective response rate of 80.3%.

The demographic characteristics of the sample show balanced gender distribution (54.4% male, 45.6% female). Most respondents were aged between 23 and 26 years. In terms of educational level, 60% were undergraduate students, 30% were master’s students, and 10% were doctoral students. The sample represented students from 42 different countries, including Asia (41.1%), Africa (24.4%), Europe (18.9%), and the America (15.6%). In terms of length of stay in China, 43.3% had stayed between 1 and 2 years, while 30% had stayed more than 2 years. These characteristics suggest that the sample adequately reflects the diversity of international students in China (Table 1).

**Table 1:** Demographic statistics of the sample.

Category	Item	Frequency	Percentage
Gender	Male	245	54.4%
	Female	205	45.6%
Age	18–22 years old	180	40.0%
	23–26 years old	195	43.3%
	27–30 years old	75	16.7%
National	Asia	185	41.1%
	Africa	110	24.4%
	Europe	85	18.9%
	America	70	15.6%
Monthly income	Below 2000 yuan	65	14.4%
	2000–4,000 yuan	215	47.8%
	4,000–6,000 yuan	130	28.9%
	6,000 yuan and above	40	8.9%
Education	Degree	270	60.0%
	Master Degree	135	30.0%
	PhD	45	10.0%
Duration in China	Below 1 year	120	26.7%
	1–2 years	195	43.3%
	2 years and above	135	30.0%

### 3.2 Measuring instruments

All measurement items used in this study were adapted from established and validated scales in prior literature to ensure reliability and validity. All constructs were measured using a five-point Likert scale ranging from 1 (strongly disagree) to 5 (strongly agree), measurement items refer to the Appendix I.

#### 3.2.1 Social media use

Social media use refers to the extent to which international students engage with social media platforms in their daily lives. The measurement items were adapted from the Facebook Intensity Scale developed by Hughes et al. (2012). Considering the Chinese social media environment, Facebook was replaced with commonly used platforms such as WeChat, Tiktok, and Xiaohongshu. The scale included four items measuring frequency of use, duration of use, and emotional dependence.

#### 3.2.2 Perceived value

Perceived value in the consumption environment is conceptualized as consisting of two key components which are product value and service value. Product value refers to the international student’s overall evaluation of the utility of products in the host country’s marketplace. Drawing on the PERVAL scale developed by Sweeney and Soutar (2001) and the value conceptualization of Zeithaml (1988). Product value was measured using six items that capture two underlying sub-dimensions which are functional value and symbolic value.

Service value refers to the international student’s evaluation of the utility derived from service encounters in the host country. Based on the service quality and value frameworks proposed by Parasuraman et al. (1988) and Brady and Cronin (2001). Service value was measured using six items that reflect two distinct sub-dimensions which are efficiency and cultural friendliness. The distinction between efficiency and cultural friendliness is particularly relevant in cross-cultural consumption contexts, where international students may value not only prompt and reliable service but also welcoming and culturally accommodating interactions.

Thus, product value focuses on what is purchased (goods), while service value focuses on the process of delivery and interpersonal interactions during consumption. All 12 items were adapted to the specific context of international students in China.

#### 3.2.3 Cross-cultural consumption adaptation

Cross-cultural consumption adaptation refers to the process by which international students adjust their consumption cognition, emotions, and behaviors to adapt to the Chinese consumption environment. This construct was adapted from the sociocultural adaptation scale developed by Ward and Kennedy (1999), contextualized within consumer acculturation theory (Berry, 1997; Cleveland and Laroche, 2007). The scale includes three dimensions: cognitive adaptation, emotional adaptation, and behavioral adaptation, measured using six items.

#### 3.2.4 Emotional value

Emotional value refers to the affective benefits that consumers obtain from consumption experiences, such as pleasure, enjoyment, excitement, or relaxation. The measurement items were adapted from Sweeney and Soutar (2001) and the consumption value theory of Sheth et al. (1991). Emotional value was measured using four items capturing the extent to which international students experience positive emotional states, for example, feeling happy, relaxed, or satisfied when engaging with Chinese products and services.

#### 3.2.5 Cultural identity

Cultural identity refers to the degree to which international students feel a sense of belonging and emotional attachment to Chinese culture. The measurement six items were adapted from the Multigroup Ethnic Identity Measure (MEIM) proposed by Phinney et al. (2001). Cultural identity was measured through cognitive identity, emotional identity, and behavioral identity.

#### 3.2.6 Purchase intention

Purchase intention represents the likelihood that international students will purchase Chinese domestic brands or culturally distinctive Chinese products. The scale was adapted from the purchase intention scale developed by Dodds et al. (1991), consisting of five items measuring purchase likelihood, purchase planning, and recommendation intention.

To ensure linguistic equivalence, the questionnaire was translated using the back-translation method. First, the original English items were translated into Chinese. Then, a second bilingual researcher translated the Chinese version back into English (Brislin, 1970). Finally, experts in cross-cultural research reviewed the translations to ensure semantic equivalence.

### 3.3 Common method bias

Because all data were collected via self-reported questionnaires, common method bias (CMB) could potentially inflate the observed relationships. To minimize this risk, procedural and statistical remedies were implemented following the recommendations of Podsakoff et al. (2012). Procedurally, respondents were assured of anonymity and confidentiality, item order was randomized, reverse-coded items were included, and the measurement of predictor and criterion variables was temporally separated. Statistically, a single-factor confirmatory factor analysis (CFA) comparison approach was employed, which is more rigorous than the traditional Harman test. Specifically, the theoretical seven-factor measurement model (where each item loads only on its intended construct) was compared against a single-factor model (where all 37 items were forced to load on one common factor). The seven-factor model exhibited excellent fit: χ^2^/df = 1.567, CFI = 0.959, TLI = 0.953, RMSEA = 0.038, SRMR = 0.044. In contrast, the single-factor model produced dramatically worse fit: χ^2^/df = 6.184, CFI = 0.623, TLI = 0.601, RMSEA = 0.109, SRMR = 0.158, see Table 2. The ΔCFI between the two models is 0.336, which far exceeds the recommended threshold of 0.10 (Podsakoff et al., 2012), indicating that a single common factor cannot account for the covariance structure of the data. These findings suggest that common method bias does not pose a serious threat to the validity of our conclusions.

**Table 2:** Comparison of model fit for common method bias test.

Model	χ^2^/df	CFI	TLI	RMSEA	SRMR
Seven-factor model (theoretical)	1.567	0.959	0.953	0.038	0.044
Single-factor model	6.184	0.623	0.601	0.109	0.158
Recommended threshold	<3	>0.90	>0.90	<0.08	<0.08
Difference	4.617	0.336	0.352	0.071	0.114

The ΔCFI of 0.336 > 0.10 indicates that common method bias is unlikely to be a substantive concern.

### 3.4 Data analysis method

Data analysis was conducted using SPSS 26.0 and AMOS 24.0. The analysis followed several steps. First, descriptive statistical analysis was performed to examine the distribution of demographic variables and the mean and standard deviation of the main constructs. Second, reliability and validity tests were conducted to assess the quality of the measurement scales. Internal consistency reliability was evaluated using Cronbach’s alpha and composite reliability (CR), with values above 0.70 indicating acceptable reliability (Hair et al., 2010). Convergent validity was assessed using factor loadings and average variance extracted (AVE), where AVE values above 0.50 are considered acceptable (Fornell and Larcker, 1981). Discriminant validity was assessed by comparing the square root of AVE with inter-construct correlations. In addition, the HTMT (heterotrait-monotrait) ratio was calculated following Henseler et al. (2015) to provide a more rigorous test of discriminant validity. Third, structural equation modelling (SEM) was employed to test the proposed research model. The analysis followed a two-step approach suggested by Anderson and Gerbing (1988). In the first step, confirmatory factor analysis (CFA) was conducted to evaluate the measurement model. In the second step, the structural model was estimated to test the hypothesized relationships among constructs. Model fit was evaluated using multiple indices, including the chi-square/degrees of freedom ratio (χ^2^/df), comparative fit index (CFI), Tucker–Lewis index (TLI), root mean square error of approximation (RMSEA), and standardized root mean square residual (SRMR). Generally, χ^2^/df values below 3, CFI and TLI values above 0.90, and RMSEA and SRMR values below 0.08 indicate acceptable model fit. Forth, mediation effects were tested using the bootstrap method with 5,000 resamples, following the procedure recommended by Hayes and Scharkow (2013). Fifth, moderation effects were tested using hierarchical multiple regression with SPSS 26.0, because testing latent variable interactions within SEM (e.g., using product-indicator or orthogonalization methods) requires specialized procedures (Marsh et al., 2004). Factor scores for product value, service value, and social media use were computed by averaging their respective item scores, justified by the high factor loadings and composite reliabilities obtained from the measurement model. Following Cohen et al. (2013), all independent variables were mean-centered before creating interaction terms. For each dependent variable (cross-cultural consumption adaptation and emotional value), we entered the main effects in Step 1 and the interaction term (e.g., centered product value × centered social media use) in Step 2. A significant change in *R*^2^ (ΔR^2^) and a significant interaction coefficient were interpreted as evidence of moderation. Simple slopes were calculated at one standard deviation above and below the mean of the moderator (social media use).

## 4 Result

### 4.1 Measurement model

The measurement model was evaluated using reliability, convergent validity, and discriminant validity following the standard procedures of structural equation modelling.

#### 4.1.1 Reliability

Internal consistency reliability was assessed using Cronbach’s alpha. The results indicate that all constructs demonstrate high reliability, with Cronbach’s alpha values ranging from 0.857 to 0.921, exceeding the recommended threshold of 0.70, see Table 3. Specifically, purchase intention exhibits the highest reliability (*α* = 0.921), followed by cultural identity (*α* = 0.914), cross-cultural consumption adaptation (*α* = 0.897), product value (α = 0.886), emotional value (*α* = 0.879), service value (*α* = 0.871), and social media use (*α* = 0.857). These results confirm that all measurement scales possess strong internal consistency.

**Table 3:** Reliability test results.

Variables	Questions	Cronbach’s *α*	Evaluation
Product value	6	0.886	Good
Service value	6	0.871	Good
Cross-cultural consumption adaptation	6	0.897	Good
Emotional value	4	0.879	Good
Cultural identity	6	0.914	Excellent
Social media use	4	0.857	Good
Purchase intention	5	0.921	Excellent

Notably, cross-cultural consumption adaptation, as a newly introduced construct in this study, demonstrates excellent reliability (*α* = 0.897), indicating that the adapted scale effectively captures the cognitive, emotional, and behavioral dimensions of consumption adaptation in a cross-cultural context.

#### 4.1.2 Convergent validity

Convergent validity was assessed through confirmatory factor analysis (CFA), examining factor loadings, composite reliability (CR), and average variance extracted (AVE). Table 4 showed that all factor loadings range from 0.728 to 0.852, exceeding the recommended threshold of 0.60, indicating that all items significantly load onto their respective constructs.

**Table 4:** Convergence validity test results.

Variables	Questions	Factor loadings	AVE	CR	Evaluation
Product value	PV1	0.761	0.617	0.889	Good
	PV2	0.808			
	PV3	0.789			
	PV4	0.782			
	PV5	0.794			
	PV6	0.774			
Service value	SV1	0.742	0.602	0.875	Good
	SV2	0.786			
	SV3	0.798			
	SV4	0.771			
	SV5	0.783			
	SV6	0.765			
Cross-cultural consumption adaptation	CCA1	0.818	0.643	0.899	Excellent
	CCA2	0.795			
	CCA3	0.824			
	CCA4	0.786			
	CCA5	0.819			
	CCA6	0.791			
Emotional value	EV1	0.831	0.638	0.877	Good
	EV2	0.796			
	EV3	0.768			
	EV4	0.801			
Cultural identity	CI1	0.824	0.672	0.913	Excellent
	CI2	0.845			
	CI3	0.809			
	CI4	0.794			
	CI5	0.818			
	CI6	0.828			
Social media use	SM1	0.802	0.591	0.853	Good
	SM2	0.765			
	SM3	0.728			
	SM4	0.779			
Purchase intention	PI1	0.827	0.685	0.920	Excellent
	PI2	0.852			
	PI3	0.811			
	PI4	0.824			
	PI5	0.831			

All constructs meet the recommended criteria of CR > 0.70 and AVE > 0.50. For example, cross-cultural consumption adaptation shows strong convergent validity (AVE = 0.643, CR = 0.899), while cultural identity demonstrates the highest convergent validity (AVE = 0.672, CR = 0.913). These findings confirm that the measurement model exhibits satisfactory convergent validity.

#### 4.1.3 Discriminant validity

Discriminant validity was evaluated using the Fornell–Larcker criterion. The square root of AVE for each construct is greater than its correlations with other constructs, indicating good discriminant validity.

For instance, refer Table 5, the square root of AVE for cross-cultural consumption adaptation (0.802) exceeds its correlation with cultural identity (0.58), confirming that these constructs are empirically distinct despite their theoretical relationship. Similarly, product value and service value, although moderately correlated (*r* = 0.67), remain below the threshold of multicollinearity concern, supporting their conceptual distinction. Overall, the measurement model demonstrates strong reliability and validity, providing a solid foundation for subsequent structural analysis.

**Table 5:** Discriminant validity test.

Variables	1	2	3	4	5	6	7
Product value	**0.786**						
Service value	0.67	**0.776**					
Cross-cultural consumption adaptation	0.52	0.48	**0.802**				
Emotional value	0.61	0.56	0.47	**0.799**			
Cultural identity	0.45	0.42	0.58	0.49	**0.820**		
Social media use	0.36	0.34	0.41	0.44	0.39	**0.769**	
Purchase intention	0.47	0.44	0.53	0.57	0.62	0.40	**0.828**

The bolded numbers on the diagonal represent the square root of Ave.

In addition to the Fornell-Larcker criterion, discriminant validity was assessed using the HTMT (heterotrait-monotrait) ratio as recommended by Henseler et al. (2015). As shown in Table 6, all HTMT values are below the conservative threshold of 0.85 (and also below the more lenient threshold of 0.90). The highest HTMT value is observed between product value and service value (HTMT = 0.81). Although these two constructs are moderately correlated (*r* = 0.67), the HTMT ratio remains below 0.85, supporting their empirical distinctness. Theoretically, product value captures the functional and symbolic attributes of goods, whereas service value reflects the efficiency and cultural friendliness of service encounters two related but conceptually separate dimensions of perceived value. Similarly, the HTMT between cultural identity and purchase intention is 0.74 (*r* = 0.62), well below the 0.85 threshold, confirming that although cultural identity positively influences purchase intention, the two constructs are not empirically redundant. No HTMT value exceeds 0.90, so no further remedial action is required.

**Table 6:** HTMT ratios.

Variables	1	2	3	4	5	6	7
Product Value	1.00						
Service Value	0.81	1.00					
Cross-cultural consumption adaptation	0.64	0.60	1.00				
Emotional value	0.73	0.68	0.59	1.00			
Cultural identity	0.55	0.51	0.71	0.60	1.00		
Social media use	0.44	0.42	0.50	0.53	0.48	1.00	
Purchase intention	0.57	0.54	0.64	0.70	0.74	0.49	1.00

All HTMT values are below the conservative threshold of 0.85 (Henseler et al., 2015), confirming discriminant validity. The highest value (0.81 between Product Value and Service Value) is acceptable given their theoretical distinctness.

### 4.2 Hypothesis testing

#### 4.2.1 Model fit

The structural model demonstrates excellent fit with the data. The chi-square/degrees of freedom ratio (χ^2^/df) is 1.567, well below the recommended threshold of 3. The RMSEA (0.038) and SRMR (0.044) are both below 0.08, indicating good absolute fit. Incremental fit indices are also satisfactory, with CFI = 0.959, TLI = 0.953, and NFI = 0.935, all exceeding the recommended threshold of 0.90.

These results confirm that the proposed dual-mediation and dual-moderation model fits the data well. Table 7 showed.

**Table 7:** Structural model fit index.

Fit index	Recommendation value	Actual value	Evaluation
χ^2^	–	498.37	–
df	–	318	–
χ^2^/df	<3	1.567	Excellent
RMSEA	<0.08	0.038	Excellent
SRMR	<0.08	0.044	Excellent
CFI	>0.90	0.959	Excellent
TLI	>0.90	0.953	Excellent
NFI	>0.90	0.935	Excellent
GFI	>0.90	0.912	Good
AGFI	>0.90	0.889	Close to Good
IFI	>0.90	0.960	Excellent

#### 4.2.2 Path analysis

All hypothesized direct relationships are statistically significant, providing full support for H1–H3, refer Table 8. Cultural identity (*β* = 0.406, *p* < 0.001) and emotional value (*β* = 0.348, *p* < 0.001) both significantly influence purchase intention, supporting H1 and H3. Product value (*β* = 0.304, *p* < 0.001) and service value (*β* = 0.278, *p* < 0.001) both have significant positive effects on cross-cultural consumption adaptation, supporting H2a and H2b. Similarly, product value (*β* = 0.371, *p* < 0.001) and service value (β = 0.292, *p* < 0.001) significantly influence emotional value, supporting H2c and H2d. Cross-cultural consumption adaptation has a strong positive effect on cultural identity (*β* = 0.553, *p* < 0.001), representing the strongest relationship in the model and strongly supporting H4.

**Table 8:** Structural model path coefficients.

Hypothesis	Pathway	β	S.E.	t-value	*p*-value	Result
H1	CI → PI	0.406	0.042	9.67	***	Supported
H2a	PV → CCA	0.304	0.047	6.47	***	Supported
H2b	SV → CCA	0.278	0.049	5.67	***	Supported
H2c	PV → EV	0.371	0.043	8.63	***	Supported
H2d	SV → EV	0.292	0.045	6.49	***	Supported
H3	EV → PI	0.348	0.039	8.92	***	Supported
H4	CCA → CI	0.553	0.041	13.49	***	Supported

***p < 0.001.

**Figure 2:**
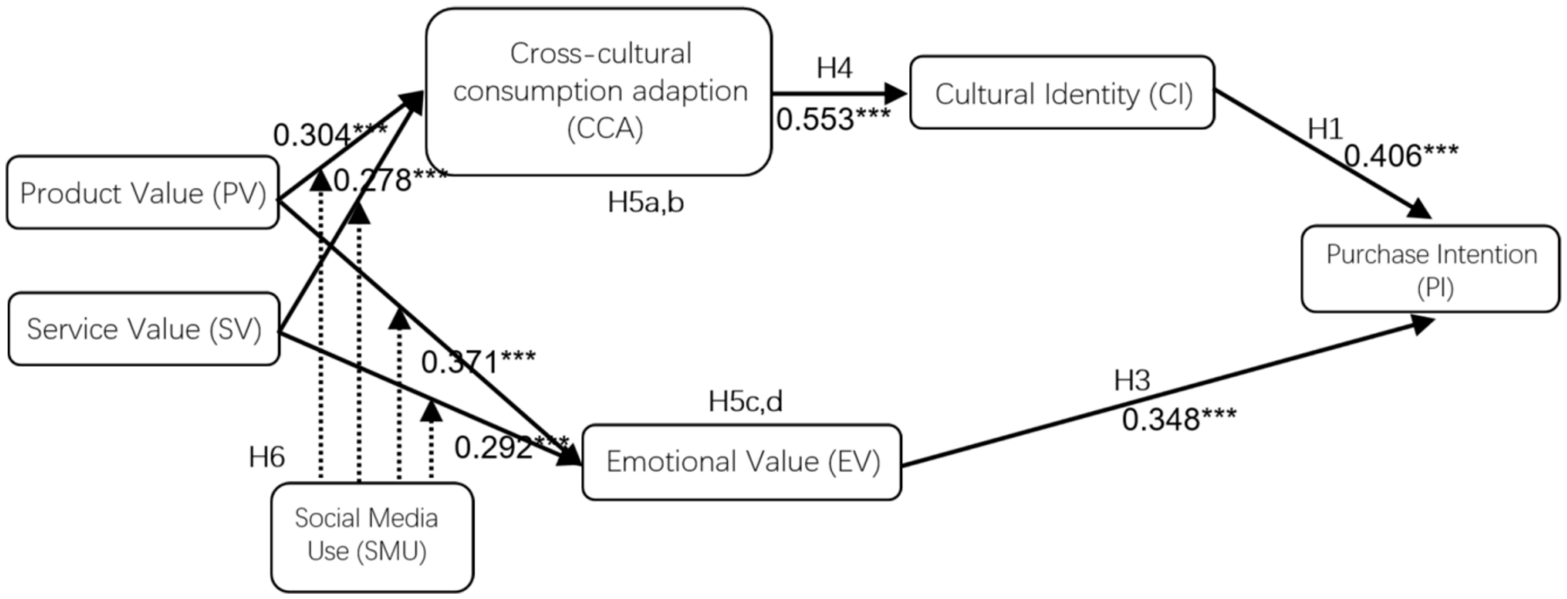
Structural model path diagram.

These findings confirm that both adaptation (adaptation → identity) and emotional (emotional value) pathways significantly contribute to purchase intention (Figure 2).

### 4.3 Mediation analysis

#### 4.3.1 Adaptation-based pathway mediation

Cross-cultural consumption adaptation significantly mediates the relationships between perceived value and cultural identity. Refer to the Table 9, the indirect effect of product value on cultural identity via adaptation is 0.168 (CI [0.122, 0.221]), accounting for 55.1% of the total effect, supporting H5a. Similarly, the indirect effect for service value is 0.154 (CI [0.109, 0.204]), accounting for 53.7% of the total effect, supporting H5b.

**Table 9:** Bootstrap test results of the mediation effect.

Hypothesis	Intermediary path	Indirect effects	Bootstrap95% Confidence interval		Proportion of total effect	Result
			Lo. limit	Up. limit		
I. The mediating role of cross-cultural consumption adaptation						
H5a	PV → CCA → CI	0.168	0.122	0.221	55.1%	Supported
H5b	SV → CCA → CI	0.154	0.109	0.204	53.7%	Supported
II. The mediating role of emotional value						
H5d	PV → EV → PI	0.129	0.094	0.170	35.6%	Supported
H5e	SV → EV → PI	0.102	0.071	0.138	31.4%	Supported

Bootstrap sampling was repeated 5,000 times at a 95% confidence level; the proportion of the total effect = (indirect effect / total effect) × 100%.

These findings indicate that adaptation is the primary mechanism through which perceived value translates into cultural identity.

#### 4.3.2 Emotional pathway mediation

Emotional value also plays a significant mediating role. The indirect effect of product value on purchase intention via emotional value is 0.129 (CI [0.094, 0.170]), accounting for 35.6% of the total effect, supporting H5c. For service value, the indirect effect is 0.102 (CI [0.071, 0.138]), accounting for 31.4% of the total effect, supporting H5d.

Comparatively, the adaptation-based pathway explains a larger proportion of the total effect (>50%) than the emotional pathway (30–36%), suggesting that cognitive adaptation and identity formation are more dominant mechanisms in cross-cultural consumption, although emotional responses remain important complementary drivers.

### 4.4 Moderation analysis

#### 4.4.1 Moderation on the adaptation-based pathway

Social media use significantly moderates the relationship between product value and cross-cultural consumption adaptation (*β* = 0.153, *p* < 0.001), supporting H6a. The interaction effect increases the explained variance (Δ*R*^2^ = 0.023). Similarly, social media use moderates the relationship between service value and adaptation (*β* = 0.138, *p* < 0.01), supporting H6b. Simple slope analysis shows that the effect of product value on adaptation is much stronger for high social media users (*β* = 0.459) than for low users (*β* = 0.176). A similar pattern is observed for service value.

#### 4.4.2 Moderation on the emotional pathway

Social media use also significantly moderates the relationship between product value and emotional value (*β* = 0.171, *p* < 0.001), supporting H6c, and between service value and emotional value (*β* = 0.159, *p* < 0.001), supporting H6d. The effect sizes are stronger under high social media usage (*β* = 0.498 vs. 0.241 for product value; *β* = 0.467 vs. 0.253 for service value), indicating a clear amplification effect.

All four moderation hypotheses are supported. Social media use strengthens both adaptation-based and emotional pathways, acting as both an information facilitator and an emotional amplifier. Its moderating effect is slightly stronger in the emotional pathway, suggesting that social media plays a particularly important role in enhancing affective experiences (Table 10).

**Table 10:** Social media moderating effect test (hierarchical regression).

Hypothesis	Pathway	Interaction term *β*	S.E.	*t*-value	Δ*R*^2^	*F* change	*p*-value	Result
H6a	PV × SMU → CCA	0.153	0.041	3.73	0.023	13.92	***	Supported
H6b	SV × SMU → CCA	0.138	0.043	3.21	0.018	10.30	**	Supported
H6c	PV × SMU → EV	0.171	0.039	4.38	0.028	19.18	***	Supported
H6d	SV × SMU → EV	0.159	0.041	3.88	0.024	15.05	***	Supported

***p < 0.001, **p < 0.01.

## 5 Discussion

### 5.1 Key findings

#### 5.1.1 The impact of perceived value on cross-cultural consumption adaptation

The empirical results demonstrate that both product value (*β* = 0.304) and service value (*β* = 0.278) exert significant and comparable effects on cross-cultural consumption adaptation. This finding confirms the applicability of perceived value theory in a cross-cultural consumption context and highlights perceived value as a critical antecedent that initiates the adaptation process. When international students perceive higher value in Chinese products and services, they are more motivated to learn and understand local consumption culture, thereby facilitating cognitive, emotional, and behavioral adaptation.

Notably, product value shows a stronger association with emotional value (*β* = 0.371) than service value (*β* = 0.292). This suggests that the symbolic and cultural attributes embedded in products such as traditional craftsmanship, aesthetic styles, and cultural meanings are more likely to evoke emotional resonance. In contrast, service value primarily generates satisfaction by reducing practical barriers, thus producing relatively weaker emotional stimulation. This distinction implies that while both product and service improvements are essential, product-related cultural symbolism may serve as a more effective driver in emotional engagement strategies.

#### 5.1.2 The mediating role of cross-cultural consumption adaptation

One of the most significant contributions of this study is the identification and validation of cross-cultural consumption adaptation as a key mediating mechanism between perceived value and cultural identity. The results indicate that the indirect effects of product value and service value on cultural identity through adaptation account for 55.1 and 53.7% of the total effects, respectively. This demonstrates that perceived value influences cultural identity primarily through a gradual adaptation process rather than a direct transition.

This finding extends cultural adaptation theory by situating it within the consumption domain. Unlike traditional applications that focus on macro-level cultural interactions, this study conceptualizes adaptation as a multidimensional process comprising cognitive, emotional, and behavioral adjustments within everyday consumption activities. It empirically confirms that consumption contexts serve as a critical arena for cultural learning and integration.

#### 5.1.3 The parallel role of emotional value

In addition to the adaptation-based pathway, emotional value functions as a parallel mediator directly linking perceived value to purchase intention. The mediation effects of emotional value account for 35.6% (product value) and 31.4% (service value) of the total effects. Although weaker than the adaptation-based pathway, the emotional pathway plays a distinct and immediate role.

This finding highlights a dual-process mechanism in cross-cultural consumption decisions. The adaptation-based pathway reflects a rational, gradual process involving cultural learning and identity construction, while the emotional pathway captures a more intuitive and immediate response driven by consumption experiences. The coexistence of these two pathways suggests that even in the absence of full cultural adaptation or identity formation, positive emotional experiences can directly stimulate purchase intention.

#### 5.1.4 The moderating role of social media use

The study confirms that social media use plays a dual moderating role in both adaptation-based and emotional pathways. On the adaptation-based side, social media strengthens the relationship between perceived value and cross-cultural consumption adaptation. For instance, the interaction effect between product value and social media use (*β* = 0.153) indicates that individuals with higher social media engagement convert perceived value into adaptation more efficiently.

On the emotional side, social media is associated with stronger emotional responses. The interaction effects (e.g., *β* = 0.171 for product value) suggest that exposure to visually rich and emotionally engaging content, such as product reviews, influencer recommendations, and user-generated feedback, is positively related to emotional value. High-frequency users are more immersed in this affective environment, making value perceptions more likely to translate into emotional value.

Thus, social media serves a dual function: as a cultural transmission channel facilitating knowledge acquisition and as an affective medium intensifying emotional experience.

### 5.2 Theoretical implications

#### 5.2.1 Extending cultural adaptation theory to consumption contexts

This study contributes to theory by extending cultural adaptation theory into the micro-level domain of consumption. By introducing and operationalizing the concept of cross-cultural consumption adaptation, it fills a gap in existing literature that largely overlooks consumption as a key context for cultural integration. The strong empirical support for this construct underscores its theoretical relevance and measurement validity.

#### 5.2.2 Enriching the explanation of cross-cultural consumer behavior

By integrating perceived value theory, cultural adaptation theory, social identity theory, and the theory of planned behavior, this study constructs a comprehensive framework featuring dual mediation and dual moderation mechanisms. The identification of both an adaptation-based pathway (adaptation–identity) and emotional pathways provides a more nuanced understanding of how purchase intentions are formed in cross-cultural settings.

Importantly, the study helps to unpack the relationship between perceived value and cultural identity by highlighting the role of cross-cultural consumption adaptation as an intermediary process, thereby addressing a gap in prior research.

#### 5.2.3 Revealing the cultural amplifier effect of social media

Another important contribution lies in identifying the role of social media in cross-cultural consumption processes. Unlike prior studies that focus on information dissemination or social interaction in isolation, this research shows that social media use is associated with both adaptation-based and emotional aspects of consumption experiences. Specifically, social media use positively moderates the relationships between perceived value and both cross-cultural consumption adaptation and emotional value, suggesting its importance in shaping how international students engage with the host country’s consumption environment.

### 5.3 Practical implications

The findings of this study offer concrete guidance for firms, universities, and policymakers seeking to facilitate international students’ cross-cultural consumption adaptation and, through it, their broader integration into the host society. Unlike prior research that examined perceived value and acculturation separately, this study demonstrates that consumption adaptation operates through two distinct yet complementary mechanisms: an adaptation-based pathway (via cross-cultural consumption adaptation) and an emotional pathway (via emotional value). Moreover, social media use strengthens both pathways. These insights provide a roadmap for designing interventions that help international students bridge cultural gaps through everyday marketplace interactions.

#### 5.3.1 For firms

First, to activate the adaptation-based pathway, firms should embed cultural symbolic value into product design and branding. For example, a local snack brand can incorporate traditional Chinese patterns or stories into packaging, helping students learn cultural meanings while consuming. Functional value (quality, reliability) must remain high, as it reduces perceived risk and encourages initial trial. Second, to enhance the emotional pathway, service providers should invest in culturally friendly service practices: training staff to show warmth, patience, and respect toward students who may struggle with language or unfamiliar customs. Multilingual signage, translation apps at service counters, and welcome gestures specifically for international students can directly boost emotional value. Third, because social media moderates both pathways, brands should leverage platforms such as Xiaohongshu and Tiktok to share user-generated content, unboxing videos, and cultural tips. A campaign featuring current international students sharing their positive consumption experiences can amplify the effects of product and service value on adaptation and emotional attachment.

#### 5.3.2 For universities

Universities play a critical role in bridging cultural adaptation through consumption. Beyond orientation lectures, hands-on marketplace orientation programs can be organized: guided trips to local supermarkets, wet markets, and shopping streets where students learn practical skills (e.g., how to use digital payment apps, interpret price tags, understand return policies). Peer mentoring schemes that pair senior international students with newcomers can provide authentic, low-anxiety learning experiences, directly facilitating cognitive and behavioral adaptation. Additionally, universities should improve on-campus consumption environments by offering multilingual service counters in canteens, bookstores, and convenience stores, as well as workshops on digital payment platforms (Alipay, WeChat Pay). These actions reduce emotional barriers (anxiety, uncertainty) and enhance emotional value, which we found to directly increase purchase intention. Finally, universities can create social media groups (WeChat, Xiaohongshu) dedicated to sharing positive consumption experiences, thereby strengthening the moderating role of social media identified in this study.

#### 5.3.3 For policymakers and host society stakeholders

Policymakers should view consumption adaptation as a lever for broader cultural integration. The study shows that when international students successfully adapt their consumption practices, they develop stronger cultural identity and higher purchase intention. This benefits local economies. To support this, policymakers can fund cultural immersion programs that include consumption-focused activities, such as traditional market visits, food festivals, and craft workshops. In these settings, students interact directly with local vendors. Policymakers can also encourage digital media initiatives, including short video challenges and cultural quizzes on Tiktok. These initiatives showcase how local products and services embody Chinese values like harmony, face, and relational ties, as discussed by Yau (1988). Policymakers should also strengthen consumer protection and service standards for cross-cultural contexts. For example, they can introduce multilingual complaint hotlines, clear price displays, and anti-discrimination guidelines for service staff. These measures increase service value, which includes efficiency and cultural friendliness. This study identifies service value as a key driver of both adaptation and emotional value. Finally, policymakers should improve payment infrastructure by simplifying the registration process for mobile wallets for international students. This reduces a major practical barrier that hinders early consumption adaptation.

### 5.4 Limitations and future research

#### 5.4.1 Limitations

This study has several limitations. First, the use of cross-sectional data limits causal inference. Therefore, the findings should be interpreted as associative rather than causal. Second, the sample is concentrated in major Chinese cities, which may affect generalizability. Third, reliance on self-reported data may introduce response biases.

#### 5.4.2 Future research directions

Future studies could adopt longitudinal designs to capture the dynamic evolution of adaptation and identity. Experimental methods may help establish causal relationships more rigorously. Additionally, extending the model to other cross-cultural groups and consumption contexts would further validate its applicability and robustness.

Overall, this study provides a comprehensive and theoretically grounded explanation of cross-cultural consumption behavior, highlighting the importance of cross-cultural consumption adaptation, the presence of dual pathways, and the moderating role of social media in shaping international students’ purchase intentions.

## Data Availability

The original contributions presented in the study are included in the article/Supplementary material, further inquiries can be directed to the corresponding author/s.

## Footnotes

1 Ministry of Education of the People’s Republic of China http://www.moe.gov.cn/jyb_xxgk/xxgk/neirong/tongji/jytj_lxsj/.
