# Supplementary material for: Bridging cultural gaps through consumption: how social media and perceived value shape international students’ purchase intentions in China
Source: Front Psychol. 2026 May 29;17:1841619. doi: 10.3389/fpsyg.2026.1841619 (PMC13260715; doi:10.3389/fpsyg.2026.1841619)
Supplement: Supplementary file 1 [file Supplementary_file_1.docx]

**Appendix I: Measurement Items and Sources**

| Variable | Measurement Items | References |
| --- | --- | --- |
| ****Social Media Use**** | 1. I check social media multiple times every day. 2. I spend a lot of time on social media. 3. Social media has become part of my daily routine. 4. I often browse content about China on social media. | Hughes et al. (2012) |
| ****Product Value**** | 1. Chinese products have good quality. 2. The performance of Chinese products meets my needs. 3. Chinese products offer good value for money. 4. Using Chinese products reflects my taste. 5. Using Chinese products helps me feel more integrated into Chinese society. 6. Chinese products have unique cultural connotations. | Sweeney and Soutar (2001); Zeithaml (1988) |
| ****Service Value**** | 1. Services in China have fast response times. 2. Mobile payment and other services in China are very convenient. 3. The overall efficiency of consumption in China is high. 4. Service staff are friendly to international students. 5. I can feel cultural inclusiveness during service interactions. 6. Services in China take into account the special needs of international students. | Parasuraman et al. (1988); Brady and Cronin Jr (2001) |
| ****Cross-Cultural Consumption Adaptation**** | 1. I understand the purchasing habits of Chinese consumers. 2. I am familiar with major shopping platforms and methods in China. 3. I feel comfortable shopping in China. 4. Consuming in China makes me feel happy. 5. I have gotten used to Chinese consumption methods (e.g., mobile payment). 6. I actively learn and try new consumption models in China. | Berry (1997); Cleveland and Laroche (2007) |
| ****Emotional Value**** | 1. Shopping in China makes me feel delighted. 2. Buying Chinese products or services makes me feel satisfied. 3. My consumption experience in China makes me feel excited. 4. Chinese consumption culture brings me positive emotional experiences. | Sweeney and Soutar (2001); Sheth et al. (1991) |
| ****Cultural Identity**** | 1. I have a clear understanding of Chinese culture. 2. I can understand the values of Chinese culture. 3. I feel proud to experience Chinese culture. 4. I have developed a sense of belonging to Chinese culture. 5. I actively practice Chinese cultural customs in daily life. 6. I am willing to introduce and recommend Chinese culture to others. | Phinney et al. (2001) |
| ****Purchase Intention**** | 1. I am willing to purchase Chinese domestic brand products. 2. I plan to continue using Chinese products or services in the future. 3. Even after returning to my home country, I will consider buying Chinese brands. 4. I will recommend Chinese products or services to my friends. 5. If the price is reasonable, I am very likely to purchase Chinese products. | Dodds et al. (1991) |
